# Stimulatory G-Protein *α* Subunit Modulates Endothelial Cell Permeability Through Regulation of Plasmalemma Vesicle-Associated Protein

**DOI:** 10.3389/fphar.2022.941064

**Published:** 2022-06-03

**Authors:** Lifan He, Hanlin Lu, Xuyang Ji, Jianying Chu, Xiaoteng Qin, Min Chen, Lee S. Weinstein, Jiangang Gao, Jianmin Yang, Qunye Zhang, Cheng Zhang, Wencheng Zhang

**Affiliations:** ^1^ The Key Laboratory of Cardiovascular Remodeling and Function Research, Chinese Ministry of Education, Chinese National Health Commission and Chinese Academy of Medical Sciences, The State and Shandong Province Joint Key Laboratory of Translational Cardiovascular Medicine, Department of Cardiology, Qilu Hospital, Cheeloo College of Medicine, Shandong University, Jinan, China; ^2^ Cardiovascular Disease Research Center of Shandong First Medical University, Central Hospital Affiliated to Shandong First Medical University, Jinan, China; ^3^ Department of Obstetrics and Gynecology, Qilu Hospital, Shandong University, Jinan, China; ^4^ Metabolic Diseases Branch, National Institute of Diabetes, Digestive, and Kidney Diseases, National Institutes of Health, Bethesda, MD, United States; ^5^ School of Life Science and Key Laboratory of the Ministry of Education for Experimental Teratology, Shandong University, Jinan, China

**Keywords:** endothelial permeability, Gsα, CREB, PLVAP, edema

## Abstract

Endothelial cell leakage occurs in several diseases. Intracellular junctions and transcellular fashion are involved. The definite regulatory mechanism is complicated and not fully elucidated. The alpha subunit of the heterotrimeric G-stimulatory protein (Gsα) mediates receptor-stimulated production of cyclic adenosine monophosphate (cAMP). However, the role of Gsα in the endothelial barrier remains unclear. In this study, mice with knockout of endothelial-specific Gsα (Gsα^ECKO^) were generated by crossbreeding Gsα^flox/flox^ mice with Cdh5-CreER^T2^ transgenic mice, induced in adult mice by tamoxifen treatment. Gsα^ECKO^ mice displayed phenotypes of edema, anemia, hypoproteinemia and hyperlipoproteinemia, which indicates impaired microvascular permeability. Mechanistically, Gsα deficiency reduces the level of endothelial plasmalemma vesicle-associated protein (PLVAP). In addition, overexpression of Gsα increased phosphorylation of cAMP response element-binding protein (CREB) as well as the mRNA and protein levels of PLVAP. CREB could bind to the CRE site of PLVAP promoter and regulate its expression. Thus, Gsα might regulate endothelial permeability *via* cAMP/CREB-mediated PLVAP expression.

## Introduction

The endothelial barrier plays a crucial role in organ function. Dysregulated endothelial permeability contributes to many pathological progresses and affects the treatment of diseases. Transendothelial fluid sieving is controlled by the vascular barrier and hydrostatic, oncotic forces that drive movement across the endothelium, as described by the Starling equation (Claesson-Welsh et al., 2021; Michel, 1997). Increased endothelial permeability is a prominent feature of many diseases such as asthma, arthritis, chronic bowel disease, cancer, infections, trauma, ischemic stroke, and other conditions where leakage due to increased endothelial permeability could result in edema, impaired function and morbidity (Middleton et al., 2004; Sandoval and Witt, 2008; Tomita et al., 2021). Intracellular junctions connect adjacent endothelial cells and govern the extravasation of plasma and the exchange of its macromolecular constituent. Some extravasation processes occur in a transcellular fashion. Solutes or cells are taken up by endothelial cells and are transported within a vesicle-like structure from the luminal to the abluminal side under inflammatory conditions (Wettschureck et al., 2019). These vesicle-like structures are usually caveolae and vesicular-vacuolar organelle (VVO) (Dvorak and Feng, 2001). Plasmalemma vesicle-associated protein (PLVAP) is an endothelial cell-specific single-span, type II membrane glycoprotein and forms homodimers *in situ* (Stan et al., 1999a; Stan, 2004). This membrane protein is involved in the formation of the diaphragms that bridge endothelial fenestrae (Stan et al., 1999b). In some organs, such as the lung, PLVAP is required for the formation of caveolae stomata and transendothelial channels (Simionescu et al., 1983). Homozygous stop mutation of Plvap gene in a newborn cause fetal protein-losing enteropathy (PLE) (Broekaert et al., 2018). Homozygous disruption of the Plvap gene in a mixed background leads to growth retardation and decreased survival. The organs with fenestrated capillaries in PLVAP knockout mice showed diaphragm disappearance of caveolae, transendothelial channels (TEC) and fenestrate causing hypoproteinemia and compromising endothelial barrier function. Endothelial PLVAP specifically germline deletion mice had similar features to global PLVAP knockout mice (Stan et al., 2012). Besides, endothelial cell-specific deletion of PLVAP in adult mice induced by tamoxifen were also observed with the loss of plasma protein from circulation and edema formation in multiple organs. Simultaneously, LPS/endotoxin-induced lung injury increased lung endothelial permeability through PLVAP (Jones et al., 2020). Thus, PLVAP plays an indispensable role in endothelial barrier function. However, the regulation of PLVAP expression remains unclear.

The alpha subunit of the stimulatory G protein (Gsα) is expressed in many cell types and is responsible for receptor-stimulated cyclic adenosine monophosphate (cAMP) generation (Weinstein et al., 2007). Gsα signaling has been revealed to play significant roles in skeletal development, neurite formation, neurobiology of learning and memory, inflammatory reactions, tumorigenesis, and others (Cong et al., 2019; Dimitrov et al., 2019; Kelly et al., 2008; Rao et al., 2016; Sarma et al., 2015). We previously reported that smooth muscle-specific Gsα deletion mice displayed severe intestinal obstruction due to decreased contractility of the intestinal smooth muscle (Qin et al., 2017). Moreover, Gsα deficiency in smooth muscle cells promoted smooth muscle phenotype switching and exaggerated angiotensin II-induced abdominal aortic aneurysm formation in mice (Qin et al., 2019). However, the role of Gsα in regulating endothelial cell homeostasis and permeability remains unknown.

In this study, we explored the function of endothelial Gsα *in vivo* using endothelial-specific Gsα knockout mice and found that Gsα deficiency caused many phenotypes such as edema and hypoproteinemia.

## Materials and Methods

### Generation of Endothelial-Specific Gsα Knockout Mice

All mice comprised of a C57BL/6J genetic background. The endothelial-specific Gsα knockout (Gsα^ECKO^) mice were generated as follows. Gsα^flox/flox^ mice (Chen et al., 2005) were crossbred with transgenic mice expressing CreER^T2^ under the control of the Cdh5 promoter (Wang et al., 2010). Recombination was induced in six-week-old mice by daily intraperitoneal administration of tamoxifen (Sigma-Aldrich, St. Louis, MO, United States) dissolved in ethanol: corn oil (1:10) solution with the dose of 1 mg/d for 5 consecutive days. Gsα^flox/flox^/Cre-mice were used as control (CTR) for all experiments. Mice were housed in individually ventilated cages under standard housing conditions (22°C, 12 h light/dark cycle), with ad libitum access to chow a diet and water. All animal procedures were approved by and conducted in accordance with the National Institutes of Health Guidelines and with the approval of the Animal Care and Use Committee of Shandong University (Approval No. DWLL-2018-018).

### Blood Pressure Measurement

The heart rate (HR), systolic, diastolic and mean blood pressures of conscious adult mice were recorded indirectly and noninvasively using a tail-cuff system (BP-2010E; Softron, Tokyo, Japan). After the mice were placed in a hop pocket, the sensor was positioned at the base of the tail. The pocket was kept in a prewarmed box at 37°C, and blood pressure was measured for 20 min at the same time every day. Animals were acclimated to the system for 7 consecutive days before blood pressure measurement.

### Evans Blue Dye Extravasation Assay.

CTR and Gsα^ECKO^ mice underwent Evans blue dye extravasation assay as described (Sangwung et al., 2017). EB dye was obtained from Solarbio (Beijing, China). Male CTR and Gsα^ECKO^ mice were injected intravenously with 20 mg/kg body weight of sterile EB dye. Mustard oil diluted to 5% in mineral oil was applied to the dorsal and ventral surfaces of the ear using a cotton swab, which was repeated after 15 min. Mice were anesthetized with ketamine and xylazine (100 mg/kg+5 mg/kg respectively, i.p.) and photographs were taken 30 min after the injection of EB dye. The whole body was perfused with phosphate buffer saline (PBS), and the lung, heart, kidney, liver, and skin were harvested. The EB dye was extracted from the organs with 1 ml of formamide overnight at 55°C and measured spectrophotometrically at 600 nm. The amount of EB extracted in formamide was calculated against a standard curve of known EB concentrations.

### Transmission Electron Microscopy

Mice were anesthetized with ketamine and xylazine (100 mg/kg+5 mg/kg respectively, i.p.). Organs were harvested, fixed in 2.5% glutaraldehyde/4% paraformaldehyde fixative, and then cut in 1 mm^3^ blocks. The blocks were then rinsed with 0.1 M sodium cacodylate and postfixed in 1% OsO_4_/0.1 M sodium cacodylate. Then the blocks were briefly rinsed, stained with Kellenberger’s uranyl acetate, dehydrated through graded ethanol and embedded in Epon812 resin (Sigma-Aldrich). Ultrathin sections (20–40 nm) were cut and stained with uranyl acetate and lead citrate. The sections were examined with JEOL-1200EX using a bottom-mount MORADA-G2 camera.

### Blood Sampling and Biochemical Analysis

The blood was isolated and immediately collected in microcontainer tubes coated with clotting activators containing a gel separator (BD Biosciences, Plymouth, United Kingdom). Blood was allowed to clot for at least 30 min before serum separation by centrifugation at 3,000 rpm for 15 min. The biochemical analysis was performed using the automatic biochemical analyzer (Chemray 240) to measure albumin, alanine aminotransferase (ALT), aspartate aminotransferase (AST), blood urea nitrogen (BUN), creatinine, urine protein, high-density lipoprotein (HDL), low-density lipoprotein (LDL), triglycerides (TC), and total cholesterol (TG) levels. For routine blood tests, the blood was isolated and immediately collected in EDTA-coated microtainer tubes (BD Biosciences). Blood cell compositions were analyzed using a ProCyte Dx Hematology Analyzer (IDEXX, Westbrook, ME, United States) following the manufacturer’s instructions.

### Histology

The mouse organs were harvested after euthanasia, fixed in 4% formalin and embedded in paraffin. 5 μm thick sections were cut and stained with hematoxylin/eosin (H&E). Images were acquired using PRANNORAMIC SCANⅡ (3D Histech, Budapest, Hungary).

### Western Blot Analysis

Human umbilical vein endothelial cells (HUVECs) and organ tissues were homogenized in RIPA lysis buffer containing protease and phosphatase inhibitors. Lysates were separated by SDS-PAGE and transferred to PVDF membranes. Target proteins were probed with specific antibodies overnight and then incubated with secondary antibodies conjugated with chemiluminescent molecules. This was followed by detection of chemiluminescent reagents (Millipore, Burlington, MA, United States) using the Bio-Rad ChemDoc MP system (Bio-Rad, Hercules, CA, United States). The primary antibodies were anti-Gsα (Santa Cruz, Dallas, TX, United States), anti-CREB (Cell Signaling Technology, Boston, MA, United States), anti-phospho-CREB Ser133 (Cell Signaling Technology), anti-GAPDH (Proteintech, Chicago, IL, United States), anti-PLVAP (Proteintech), anti-Na/K ATPase (Proteintech).

### Immunofluorescent Assay

Sections underwent heat-induced epitope retrieval with sodium citrate buffer and were blocked with 10% goat serum in PBS for 1 h. The sections were incubated with anti-CD68 (Abcam, Cambridge, United Kingdom), anti-Gsα (Proteintech) and anti-CD31 (Santa Cruz) primary antibodies overnight at 4°C and goat polyclonal secondary antibody to rabbit or mouse IgG-H&L (Abcam). Finally, the sections were covered with mounting medium with DAPI (Abcam). Images were taken under a fluorescence microscope (Nikon-U, Melville, NY, United States).

### Chromatin Immunoprecipitation (ChIP) Assay

ChIP assay was performed using Simple ChIP Plus Enzymatic Chromatin IP kit (Cell Signaling Technology). Briefly, HUVECs were cross-linked with formaldehyde. DNA was sonicated according to the manufacturer’s instructions. The sheared chromatin was incubated with 1 μg rabbit IgG or anti-CREB antibody (Millipore). ChIP-DNA was purified and eluted in 50 μl of elution buffer, and 2 μl immunoprecipitated DNA was subjected to PCR using the following primers: 5′- CTT​CCC​CGC​TCC​AAG​TTC​AT -3′ and 5′- ATT​GCA​TAG​GAG​GGC​GTC​AC -3′.

### Luciferase Reporter Assay

The DNA fragment from the human PLVAP promoter extending from −1727bp to −1bp, was cloned into the pGL3 Basic vector to generate the wild-type Luc construct. The mutant construct with deletion of the CRE site in the PLVAP promoter was generated. For luciferase assay, the luciferase reporter plasmid was transfected into HEK-293T cells in 24-well plates by using lipofectamine 2000 (Thermo Fisher Scientific, Waltham, MA, United States). The p-RL-TK plasmid carrying the Renilla luciferase under control of the thymidine kinase promoter was co-transfected as an internal control. After 24 h, cells were treated with forskolin and luciferase activity was analyzed using the Dual Luciferase Assay Kit (Beyotime, Nantong, China).

### Cell Culture

HUVECs were purchased from ScienCell (Santiago, MN, United States; Lot Number 28433), and cultured in endothelial cell medium (ECM, ScienCell) supplemented with 1% endothelial cell growth supplement (ECGS, ScienCell) and 5% FBS. The culture surfaces were pre-coated by fibronectin (ScienCell) which was diluted in PBS (1:100). Cells from the fourth to sixth generations were used for the experiments. At 80%–90% confluence, HUVECs were transfected with control, Gsα, or CREB siRNA (GenePharma, Shanghai, China) using Lipofectamine RNAiMAX Transfection Reagent (Thermo Fisher Scientific). According to the manufacturer’s instructions, the culture medium was replaced with Opti-MEM (Gibco) and the RNA-lipid complexes were added to cells followed by incubating for 6 h at 37°C and replacing with normal medium. After 48 h, cells were collected for analysis. For the virus-mediated gene transduction, HUVECs were infected with adenovirus-expressing Gsα or lentivirus-expressing CREB (Vigenebio, Jinan, China) for 48 h followed by analysis. H89 and forskolin were purchased from Abcam.

### RNA Extraction and Quantitative RT-PCR

Total RNA was extracted from HUVECs using ReliaPrep RNA Cell Miniprep (Promega, Madison, Wisconsin, United States). cDNA was reversed-transcribed using the PrimeScript RT reagent kit with gDNA Eraser (Takara, Otsu, Shiga Prefecture, Japan). PCR was performed using TB Green Premix Ex Taq II (Takara) with the Roche Light Cycler 480II. The following primers were used: PLVAP: forward 5′- CCG​GGT​CAT​CTA​CAC​GAA​CA -3′ and reverse 5′- TGA​AGA​GCA​AGG​CAT​CGC​A -3′. β-actin: forward 5′-CAT​GTA​CGT​TGC​TAT​CCA​GGC-3′ and reverse 5′-CTC​CTT​AAT​GTC​ACG​CAC​GAT-3′.

### Statistical Analysis

Data were expressed as mean ± SEM and analyzed using GraphPad Prism 9 (GraphPad Software Inc., San Diego, CA, United States). Statistical comparisons of two groups using an unpaired 2-sided Student t-test. For comparisons among more than 2 groups, the One-Way ANOVA and Bonferroni post-tests were used. *p < 0.05 was considered statistically significant.

## Results

### Endothelial-Specific Deletion of Gsα in Mice Caused Edema and Impaired Postnatal Survival

**FIGURE 1:**
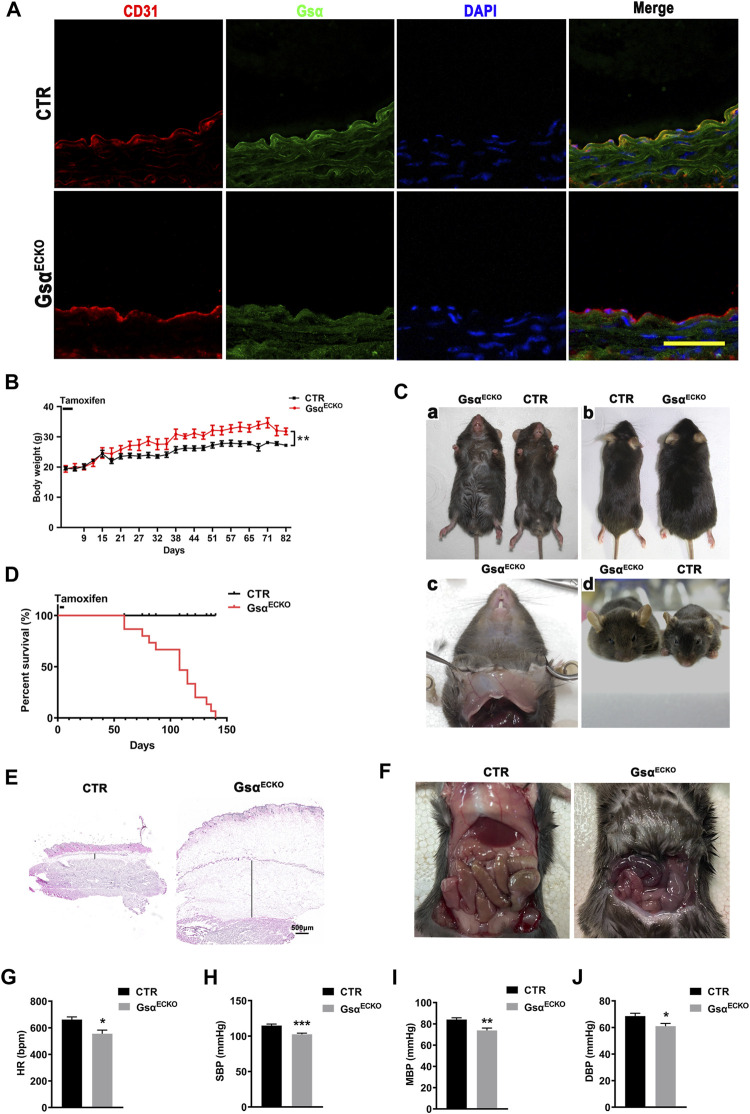
Edema and impaired postnatal survival in Gsα^ECKO^ mice. **(A)** Representative immunofluorescent staining of aortas from CTR and Gsα^ECKO^ mice to determine Gsα expression. Scale bar, 100 μm. **(B)** The growth curve for male CTR and Gsα^ECKO^ mice after tamoxifen injection, *n* = 5 per group. **(C)** Gsα^ECKO^ mice were larger than CTR **(a,b)**, transparent gelatin substance in subcutaneous tissue **(c)** and edema facial ministry **(d)**. **(D)** Kaplan Meier survival plot of CTR and Gsα^ECKO^ mice after tamoxifen injection, *n* = 15 per group. ****p < 0.0001 between 2 indicated groups by Log-rank test. **(E)** Histology of skin harvested from CTR and Gsα^ECKO^ mice. Scale bar, 500 μm. **(F)** Gsα^ECKO^ mice showed ascites, evidenced by fluid accumulation in the peritoneum of the mice. **(G)** HR (heart rate) of Gsα^ECKO^ mice and CTR, *n* = 5 per group. **(H)** Systolic pressure (SBP) of CTR and Gsα^ECKO^ mice *n* = 10 per group. **(I)** Mean blood pressure (MBP) of Gsα^ECKO^ mice and CTR, *n* = 10 per group. **(J)** Diastolic blood pressure (DBP) of Gsα^ECKO^ mice and CTR, *n* = 10 per group. Data are shown as mean ± SEM. *p < 0.05; **p < 0.01; ***p < 0.001 between 2 indicated groups by 2-tailed Student t-test.

To determine the biological significance of endothelial Gsα expression in adult mice, Gsα^flox/flox^ mice were crossbred with Cdh5-CreER^T2^ mice to generate Gsα^flox/+^/Cre + mice, which were further intercrossed to obtain Gsα^flox/flox^/Cre + mice. These mice were induced Cdh5-CreER^T2^ activity when 6 weeks old by intraperitoneal injection of tamoxifen for 5 consecutive days to delete Gsα in endothelial cells (referred to as Gsα^ECKO^ mice). The littermate Gsα^flox/flox^/Cre-mice with the same dose of tamoxifen were used as controls (CTR). Immunofluorescent assay was performed to detect Gsα expression in the aortic endothelium from CTR and Gsα^ECKO^ mice, which showed that Gsα decreased significantly in the endothelial cells of Gsα^ECKO^ mice compared with CTR (Figure 1A). The average body weight of Gsα^ECKO^ mice increased significantly at 3 weeks after tamoxifen treatment progressing in severity as the mice aged (Figure 1B). At the same time, Gsα^ECKO^ mice showed edema of lower body part primarily and spread to the upper trunk and facial ministry (Figure 1C). Transparent gelatin substance was observed in the subcutaneous tissue (Figure 1C). Staining the ventral skin with H&E, which exhibited distended a layer of subcutaneous tissue (Figure 1E). Gsα^ECKO^ mice also developed prominent pleural effusion (Figure 1F). Strikingly, endothelial Gsα deficiency mice succumbed starting 8 weeks after tamoxifen injection (Figure 1D). The HR and blood pressure of Gsα^ECKO^ mice were lower compared to those of CTR (Figures 1G–J).

### Gsα^ECKO^ Mice Increased Microvascular Permeability

**FIGURE 2:**
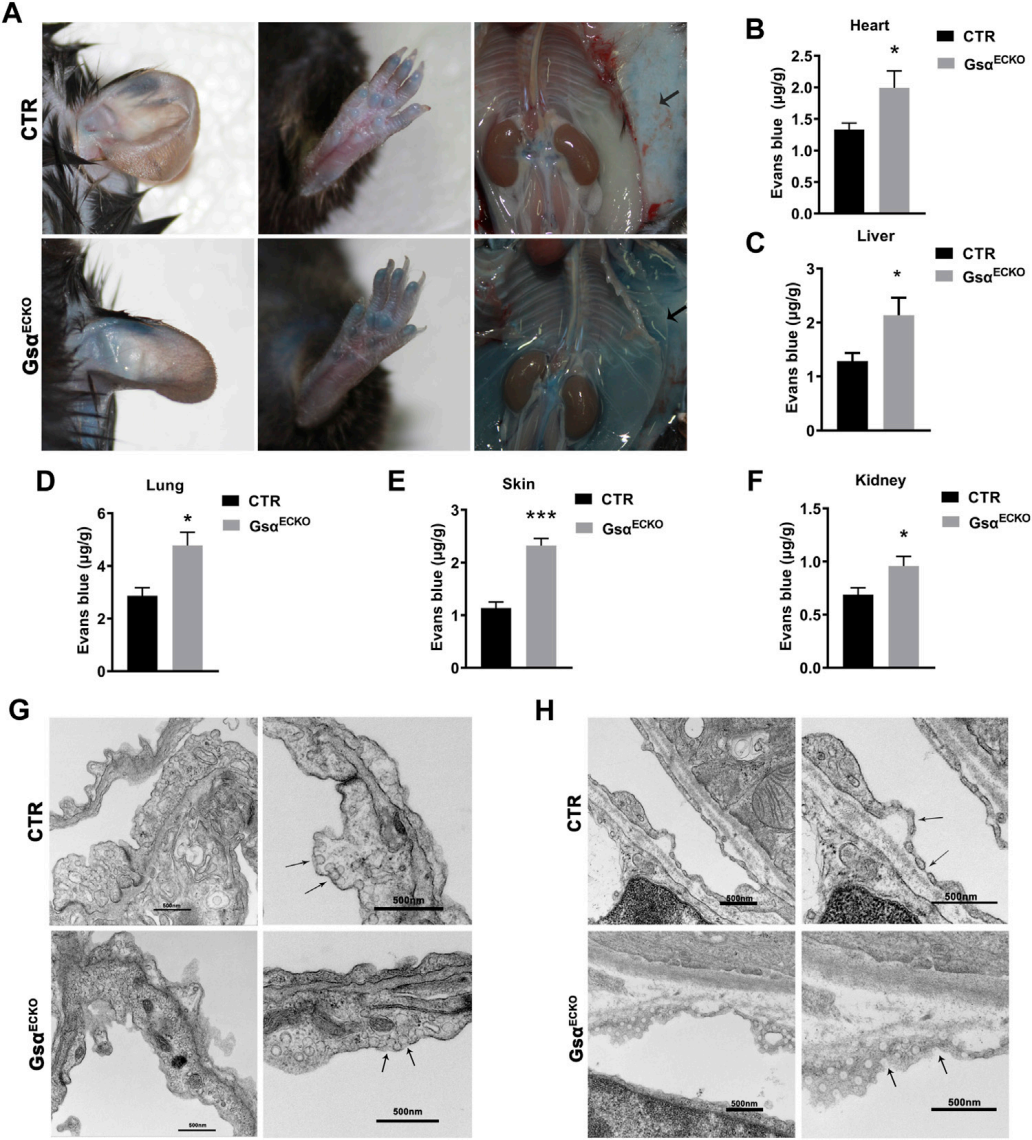
Loss of endothelial Gsα increased microvascular permeability. **(A)** Representative gross images of CTR and Gsα^ECKO^ mice after EB dye injection. The black arrow indicated EB dye exudation in Gsα^ECKO^ mice. **(B–F)** Quantification of EB dye extravasation of heart, liver, lung, skin, and kidney. *n* = 5 per group. **(G,H)** Transmission electron micrographs of capillaries from lung and kidney of CTR and Gsα^ECKO^ mice. The arrowhead indicated the absence of diaphragms. Scale bar, 50 nm. Data are shown as mean ± SEM. *p < 0.05; **p < 0.01; ***p < 0.001; ****p < 0.0001 between 2 indicated groups by 2-tailed Student t-test.

The movement of albumin from the vascular to the extravascular compartment was evaluated by using the Evans Blue (EB) dye extravasation assay. EB binds tightly to plasma proteins (especially albumin) and is normally retained in the vascular space, its extravasation indicating protein leakage into the interstitial space. Gsα^ECKO^ mice exhibited increased EB-albumin extravasation in organs with fenestrated endothelial cells especially in the skin. The appearance of Gsα^ECKO^ mice showed deeper blue in the ear, paw, subcutaneous gelatin, and perivascular adipose tissue (PVAT) (Figure 2A). Moreover, EB-albumin extravasation also increased in the lung, heart, kidney and liver (Figures 2B–F). These results suggested increased transendothelial permeability of albumin in Gsα^ECKO^ mice. Next, TEM measurement was performed to explore the ultrastructural differences. The caveolae diaphragms of lung capillaries were absent in Gsα^ECKO^ mice, while the neck and bulb diameters of the caveolae increased (Figure 2G). However, the inter-endothelial junctions appeared similar in CTR and Gsα^ECKO^ mice. In kidney peritubular capillaries, the electron opaque structure indicative of diaphragms was not found in Gsα^ECKO^ mice (Figure 2H). Thus, endothelial Gsα is necessary for the *in vivo* maintenance of capillary diaphragms and its deficiency could cause gross changes in the caveolar structure.

### Gsα^ECKO^ Mice Displayed Inflammation Infiltration in the Lung

**FIGURE 3:**
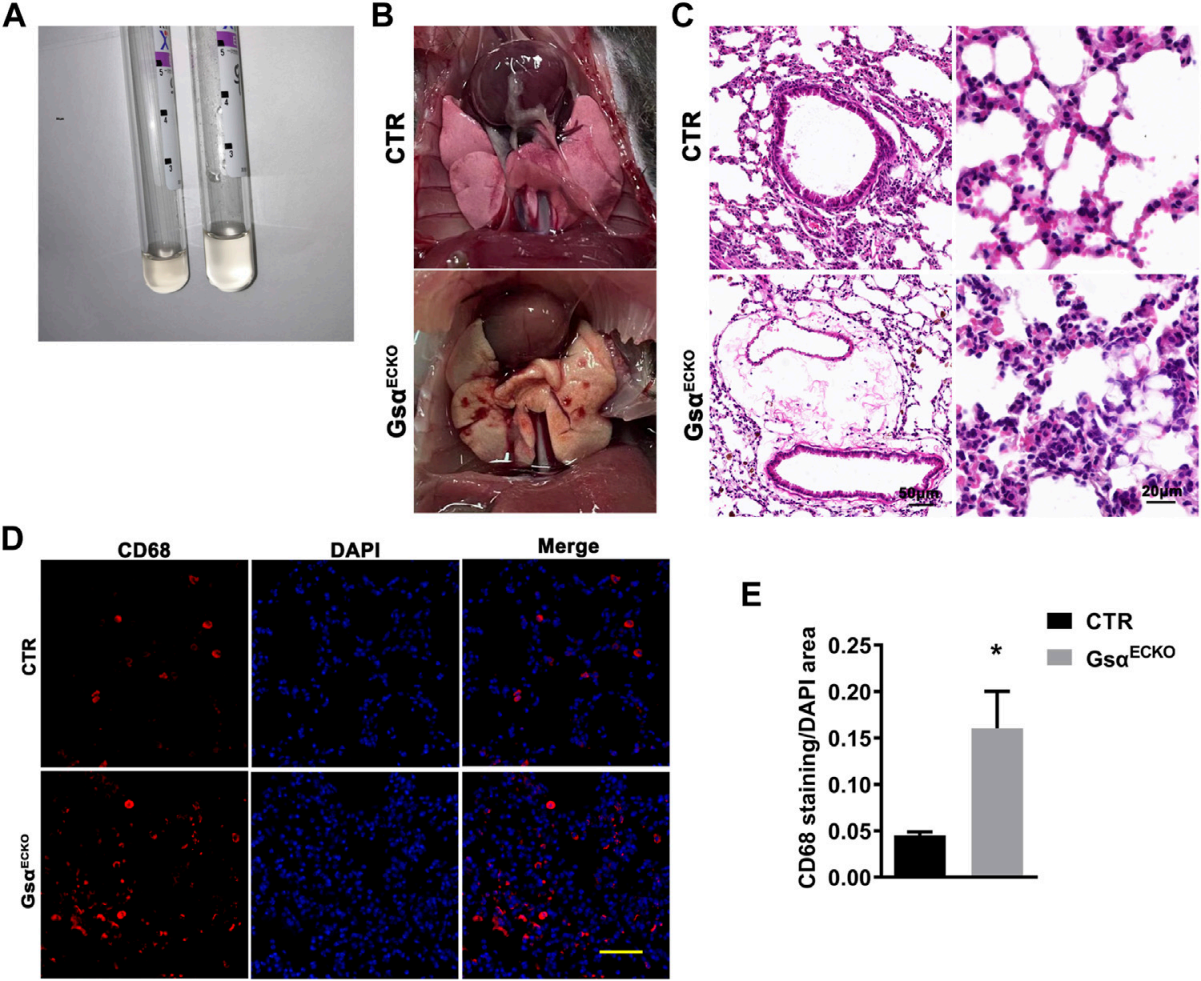
The lung tissue of Gsα^ECKO^ mice was infiltrated with inflammation. **(A)** Pleural effusion was sucked from the thoracic cavity. **(B)** Presence of petechiae on the pleural surface of Gsα^ECKO^ mice lungs. **(C)** H&E staining of lung tissue sections from CTR and Gsα^ECKO^ mice. Scale bar, 20 μm. **(D)** Representative immunofluorescent staining of CD68 in lung tissues of CTR and Gsα^ECKO^ mice. Scale bar, 100 μm. **(E)** Quantification of CD68 positive area/DAPI area. *n* = 6 to 7 per group. Data are shown as mean ± SEM. *p < 0.05 between 2 indicated groups by 2-tailed Student t-test.

Gsα^ECKO^ mice exhibited pleural effusion (Figure 3A), which coagulated into gelatin within a few minutes of being fetched. Pleural fluid contained high levels of protein and glucose, suggesting that loss of endothelial Gsα increased filtration of plasma into the extravascular spaces (Table 1). Gross examination revealed numerous petechiae on the pleural surface microvessels, indicative of red blood cells extravasation in the lung of Gsα^ECKO^ mice (Figure 3B). Vascular abnormalities were not observed in other organs examined such as kidney, liver, spleen, and brain. H&E staining showed inflammatory cell infiltration and interstitial thickening in Gsα^ECKO^ mice (Figure 3C). The immunofluorescent staining with CD68 in lung tissues indicated that macrophage infiltration increased in Gsα^ECKO^ mice (Figures 3D,E). Increasing EB-albumin extravasation and fluid extravasation reflected by H&E staining indicated that endothelial Gsα deficiency impaired endothelial barrier function especially in the lung, which led to inflammatory infiltration.

**TABLE 1:** Composition of ascites in Gsα^ECKO^ mice. Pleural fluid was collected from the thorax of Gsα^ECKO^ mice and analyzed for biochemical contents; *n* = 4.

Pleural Fluid			
	Mean	SEM	*n*
Glucose (mM)	5.4488	0.28318	4
Albumin (g/L)	10.2708	0.26769	4
Total Protein	15.9173	0.62739	4

### Loss of Endothelial Gsα Impaired Plasma Protein Homeostasis and Blood Composition

**FIGURE 4:**
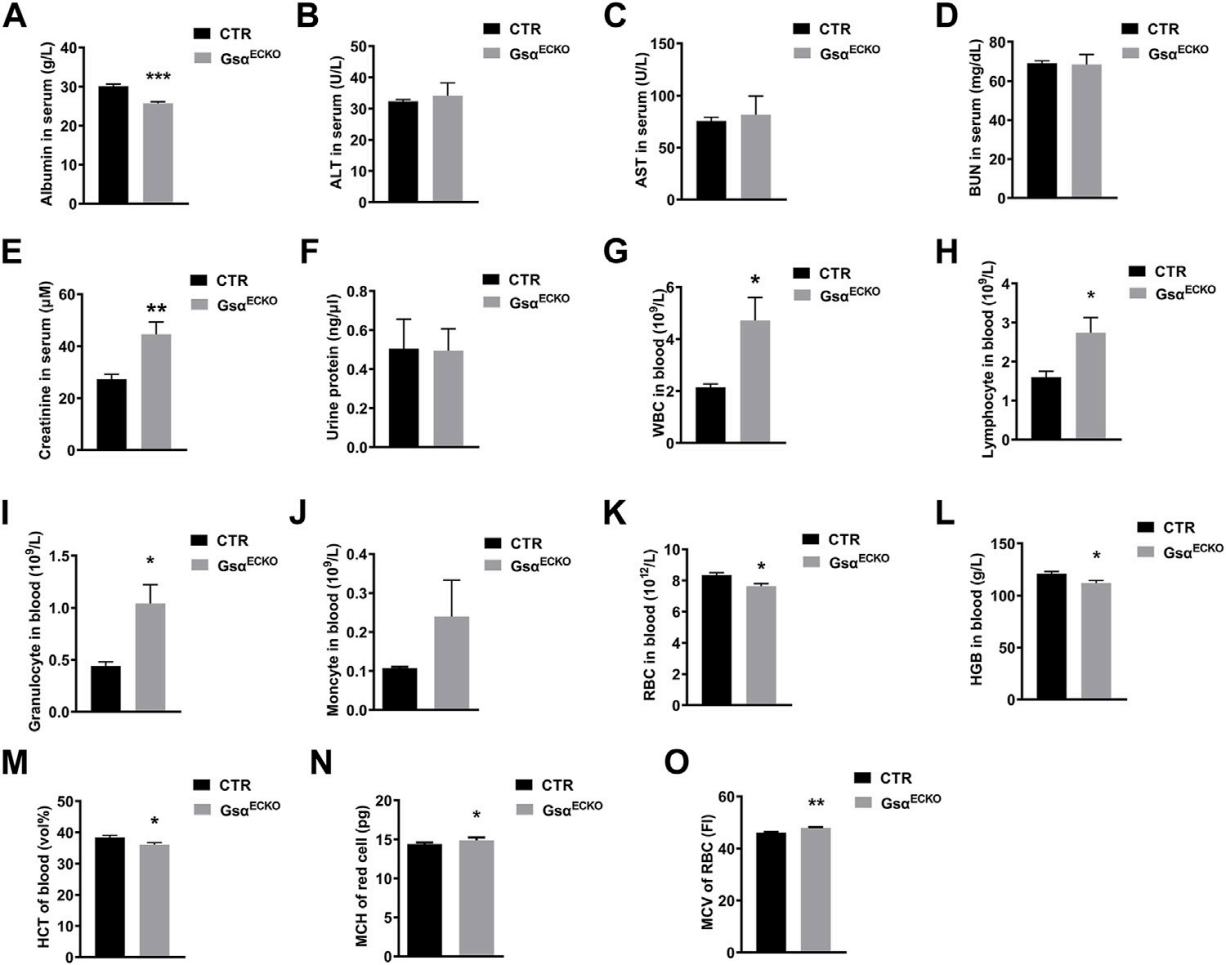
Endothelial Gsα deficiency impaired plasma protein homeostasis and disrupted blood composition. **(A–E)** Quantification of Albumin, ALT, AST, BUN, and Creatinine in blood serum of CTR and Gsα^ECKO^ mice. *n* = 5 to 6 per group. **(F)** Quantification of protein in the urine of CTR and Gsα^ECKO^ mice. *n* = 5 to 6 per group. **(G–K)** Quantification of white blood cells (WBC), Lymphocyte, Granulocyte, Monocyte, and red blood cells (RBC) in the blood of CTR and Gsα^ECKO^ mice. *n* = 5 to 6 per group. **(L–M)** Quantification of hemoglobin (HGB) and (red blood cell-specific volume) HCT in blood of CTR and Gsα^ECKO^ mice. *n* = 5 per group. **(N–O)** Mean corpuscular hemoglobin (MCH) and mean corpuscular volume (MCV) of red blood cells of CTR and Gsα^ECKO^ mice. *n* = 5 to 6 per group. Data are shown as mean ± SEM. *p < 0.05; **p < 0.01; ***p < 0.001 between 2 indicated groups by 2-tailed Student t-test.

Since endothelial Gsα deficiency in adult mice increases vascular permeability to albumin, we assessed the function of the liver and kidney. The plasma albumin level in Gsα^ECKO^ mice was significantly lower than that in CTR (Figure 4A). The serum levels of ALT, AST and BUN were similar in CTR and Gsα^ECKO^ mice (Figures 4B–D), although plasma creatinine was increased in Gsα^ECKO^ mice (Figure 4E). Gsα^ECKO^ mice exhibited normal kidney and liver histology (Supplementary Figure S1). Protein concentrations in the urine of CTR and Gsα^ECKO^ mice were not significantly different (Figure 4F). These results illustrated that endothelial Gsα deficiency does not injure the kidney and liver, so hypoproteinemia of Gsα^ECKO^ mice was not caused by abnormal liver or kidney. Routine blood test revealed that white blood cells (WBC) including lymphocytes and granulocytes were increased in Gsα^ECKO^ mice (Figures 4G–I). Monocytes had no difference between CTR and Gsα^ECKO^ mice (Figure 4J). Compared with CTR, Gsα^ECKO^ mice also exhibited significantly decreased red blood cells (RBC) and hemoglobin (HGB) levels (Figures 4K,L). Furthermore, Gsα^ECKO^ mice displayed decreased red blood cell-specific volume (HCT) (Figure 4M), increased mean corpuscular hemoglobin (MCH) and mean corpuscular volume (MCV) (Figures 4N,O). These results indicated that macrocytic anemia may occur in Gsα^ECKO^ mice.

### Knockout of Endothelial-Specific Gsα Mice Displayed Decreased Adipose Tissue

**FIGURE 5:**
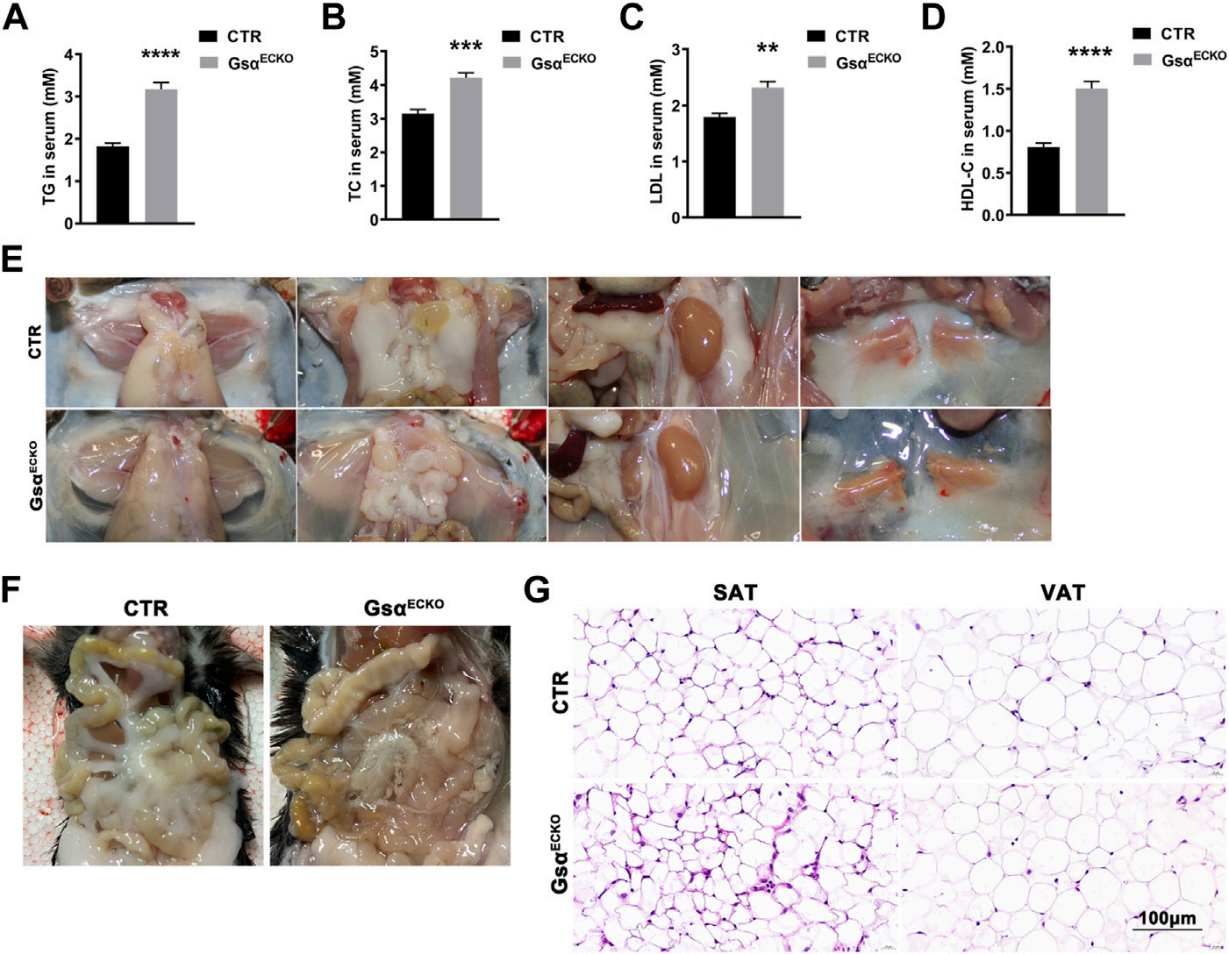
Gsα^ECKO^ mice displayed decreased blood lipid and adipose tissue. **(A–D)** Quantification of TG, TC, LDL and HDL-C in blood serum of CTR and Gsα^ECKO^ mice. *n* = 5 to 6 per group. **(E)** Representative gross anatomy images of inguinal white adipose tissue (ingWAT), epididymal white adipose tissue (eWAT), perirenal white adipose tissue (prWAT) and interscapular brown adipose tissue (isBAT) from CTR and Gsα^ECKO^ mice. **(F)** Representative gross anatomy images of mesenteric white adipose tissue (mWAT) tissue from CTR and Gsα^ECKO^ mice. **(G)** Histology of subcutaneous adipose tissue (SAT) and visceral adipose tissue (VAT) of Gsα^ECKO^ and CTR mice. Scale bar, 100 μm. Data are shown as mean ± SEM. **p < 0.01; ***p < 0.001; ****p < 0.0001 between 2 indicated groups by 2-tailed Student t-test.

Based on the previous studies, the decreasing plasma protein followed by a certain degree of hypertriglyceridemia in PLVAP^−/−^ mice (Stan et al., 2012). Serum lipid levels in Gsα^ECKO^ mice showed increased plasma TG, T-CHO, LDL, and HDL (Figures 5A–D). Although Gsα^ECKO^ mice gained more average weight than CTR at 3 weeks after tamoxifen injection, adipose tissue in Gsα^ECKO^ mice decreased. White adipose tissue (WAT) deposits in the abdominal wall, retroperitoneal, and gonadal fat pads were much less in Gsα^ECKO^ mice compared to that in CTR (Figure 5E). The inguinal white adipose tissue (ingWAT) of Gsα^ECKO^ mice showed decreased size and infiltration with gelatinous material. At the same time, mesenteric white adipose tissue (mWAT) nearly diminished in Gsα^ECKO^ mice (Figure 5F). The histological analysis of subcutaneous adipose tissue (SAT) and visceral adipose tissue (VAT) revealed no significant difference in adipocytes size between CTR and Gsα^ECKO^ mice (Figure 5G), suggesting that the production of adipocytes decreased in Gsα^ECKO^. Taken together, endothelial Gsα knockout mice not only displayed increased blood lipid but also decreased white adipose tissue deposit, which illustrated its role in the regulation of lipid metabolism.

### Gsα Deficiency Reduced Plasmalemma Vesicle-Associated Protein Expression in Endothelial Cells

**FIGURE 6:**
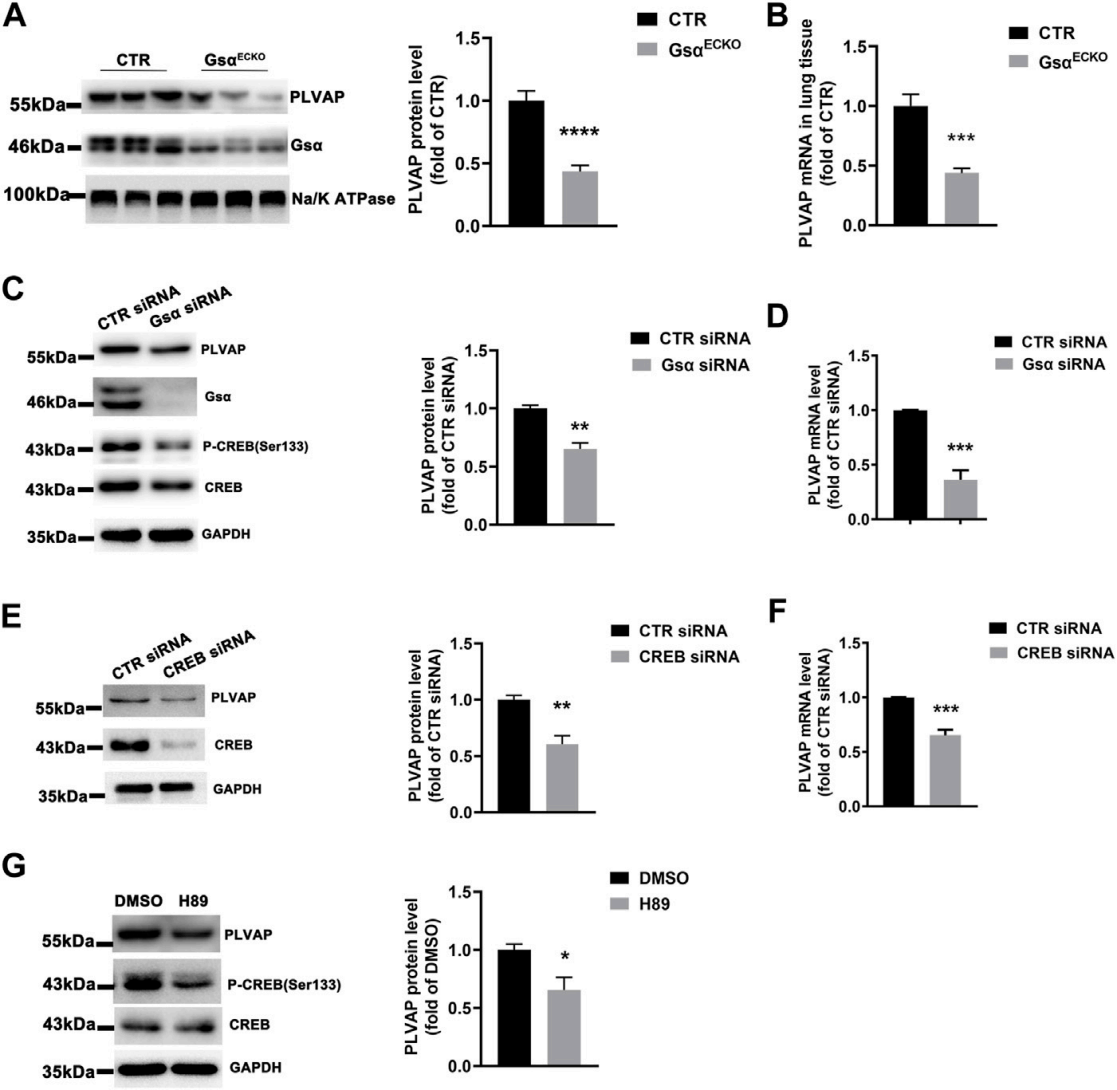
Gsα deficiency caused decreased PLVAP expression. **(A)** Western blot analysis and quantification of PLVAP level in lung membrane protein of CTR and Gsα^ECKO^ mice. *n* = 7 per group. **(B)** RT-qPCR analysis of Plvap mRNA in CTR and Gsα^ECKO^ mice tissue. *n* = 6 per group. **(C)** Western blot analysis and quantification of PLVAP in HUVECs transfected with CTR or Gsα siRNA. *n* = 3 per group. **(D)** RT-qPCR analysis of PLVAP mRNA level in HUVECs transfected with CTR or Gsα siRNA. *n* = 4 per group. **(E)** Western blot analysis and quantification of PLVAP expression in HUVECs transfected with CTR or CREB siRNA. *n* = 4 per group. **(F)** RT-qPCR analysis of PLVAP mRNA level in HUVECs transfected with CTR or CREB siRNA. *n* = 4 per group. **(G)** Western blot analysis and quantification of PLVAP in HUVECs treated with H89 (10 μM) for 24 h *n* = 3 per group. Data are shown as mean ± SEM. *p < 0.05; **p < 0.01; ***p < 0.001 between 2 indicated groups by 2-tailed Student t-test.

Gsα^ECKO^ mice displayed the phenotype of diaphragms deficiency in the lung and kidney, which was similar to that in endothelial PLVAP knockout mice. PLVAP was identified as the first known molecular component of diaphragms of fenestrated capillaries (Stan, 2004; Stan et al., 2004). Therefore, we investigated whether Gsα regulates PLVAP expression. The protein and mRNA levels of PLVAP were markedly decreased in lung tissue of Gsα^ECKO^ mice compared with CTR (Figures 6A,B). Then, knockdown of Gsα with siRNA decreased the phosphorylation of CREB and the protein and mRNA levels of PLVAP in HUVECs (Figures 6C,D). Also, CREB knockdown with siRNA suppressed PLVAP expression (Figures 6E,F). Moreover, H89, as a PKA inhibitor, inhibited CREB phosphorylation and PLVAP expression in HUVECs (Figure 6G). Thus, Gsα deficiency could decrease PLVAP expression in endothelial cells.

### Gsα Regulates PLVAP Expression *via* CREB-Mediated Transcription

**FIGURE 7:**
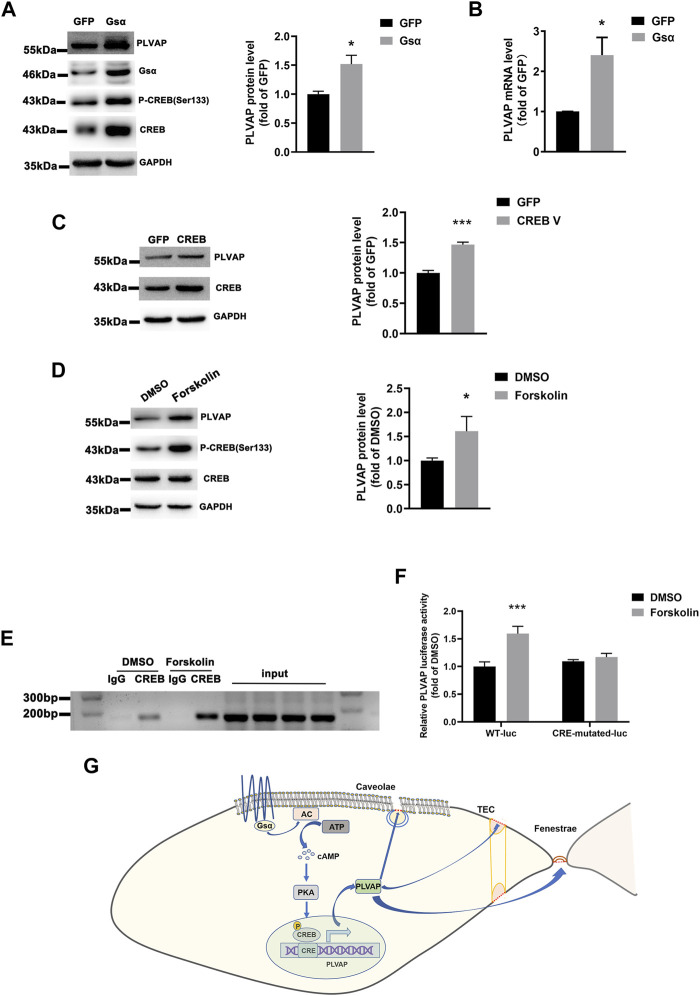
Gsα regulates PLVAP expression. **(A)** Western blot analysis and quantification of PLVAP expression in HUVECs transfected with adenovirus expressing GFP or Gsα. *n* = 5 per group. **(B)** RT-qPCR analysis of PLVAP mRNA in HUVECs infected with adenovirus-expressing GFP or Gsα. *n* = 4 per group. **(C)** Western blot analysis and quantification of PLVAP in HUVECs infected with lentivirus expressing GFP or CREB. *n* = 4 per group. **(D)** Western blot analysis and quantification of PLVAP in HUVECs treated with forskolin (10 μM) for 24 h *n* = 6 per group. **(E)** Binding of CREB to the CRE site in the PLVAP promoter was shown by Chromosome immunoprecipitation assay. **(F)** Luciferase activity in HEK-293T cells transfected with wild-type or a mutant PLVAP promoter-luciferase constructs and incubated with forskolin for 24 h. Results of luciferase promoter assay showed firefly/Renilla luciferase activity. *n* = 6 per group. Data are shown as mean ± SEM. *p < 0.05; **p < 0.01; ***p < 0.001 between 2 indicated groups by 2-tailed Student t-test. the One-Way ANOVA and Bonferroni post-tests were used in **(F)**. **(G)** The schematic diagram for the role of endothelial Gsα in regulating PLVAP expression to sustain diaphragms of fenestrated endothelium.

Gsα was overexpressed in HUVECs, and the protein and mRNA levels of PLVAP were significantly elevated (Figures 7A,B), which indicated that endothelial Gsα may contribute to the transcription of PLVAP. CREB is the Gsα/cAMP/protein kinase A (PKA)-mediated transcript factor (Zhang et al., 2020) and may regulate PLVAP expression. HUVECs infected with CREB lentivirus displayed increased PLVAP expression (Figure 7C). We also used the cAMP activator forskolin to stimulate the endothelial cells and it enhanced the PLVAP levels (Figure 7D). To determine whether Gsα regulates PLVAP expression *via* CREB-mediated transcription, the PLVAP promoter was analyzed by the Transcription Factor Database (http://jaspar.genereg.net), an Internet-based transcription-factor binding-site program, one CRE site in the PLVAP promoter was identified. To test whether CREB binds to the predicted CRE site on the PLVAP promoter, chromatin immunoprecipitation assay was performed. The result demonstrated that CREB could bind to the CRE site in the PLVAP promoter (Figure 7E). To further analyze the role of the CRE site in PLVAP promoter activity, we mutated the core CRE site of PLVAP promoter which was inserted into a luciferase plasmid and tested in HEK-293T cells. Forskolin treatment significantly increased the luciferase activity of the wild-type but not CRE mutant PLVAP promoter (Figure 7F). The results indicated that the CRE site of the PLVAP promoter is required for Gsα-induced PLVAP gene expression. Thus, our data demonstrated that Gsα regulates PLVAP expression *via* CREB-mediated transcription in endothelial cells (Figure 7G).

## Discussion

The monolayer of endothelial cells forms a semi-permeable barrier that controls substance exchange. In this study, we demonstrated that endothelial Gsα knockout mice displayed endothelial barrier impairment with increased microvascular albumin permeability and hypoproteinemia, which may contribute to the phenotype of edema and impaired postnatal survival of Gsα^ECKO^ mice. The lung tissue of Gsα^ECKO^ mice also showed numerous petechiae and inflammatory cell infiltration, accompanied by hydrothorax and ascites. In addition, Gsα^ECKO^ mice exhibited hyperlipidemia and diminished adipose tissue. Furthermore, caveolae diaphragms were absent following Gsα deficiency. Mechanistically, endothelial Gsα regulated PLVAP expression through cAMP/PKA/CREB signaling pathway.

Endothelial cells are highly metabolically active and play a critical role in regulating visuomotor tone, permeability, angiogenesis and both innate and adaptive immunity, which has been recognized in most diseases (Aird, 2007). Endothelial cell heterogeneity refers to phenotypic diversity across the vascular tree. The exchange of substances between blood and underlying tissue takes place primarily in capillaries. When endothelium is exposed to inflammatory cytokines and other factors, vascular permeability increases and plasmid fluid as well as proteins extravasate, which could sustain under chronic inflammation and cancer (McDonald, 2001). Usually, the transportation of fluid and small solutes occurs in paracellular manners, while macromolecules are transferred through caveolae VVOs and transendothelial channels. Caveolae and fenestrae possess a thin non-membranous stomatal diaphragm that contains the PLVAP protein, which is a key mediator in transcytosis of albumin across endothelial cells, inflammation-induced permeability, and leukocyte migration (Zhao and Zhao, 2020). As shown in this study, endothelial Gsα knockout mice revealed significant similarity to PLVAP knockout mice, which suffered from edema, anemia, and hyperlipoproteinemia although its severity varied with the background and strain of the mice (Jones et al., 2020; Stan et al., 2012).

PLVAP expression could be modulated by several compounds, signaling molecules, and biological processes. Vascular endothelial growth factor (VEGF) could regulate PLVAP expression in a phosphatidylinositol 3-kinase (PI3K)- or p38 mitogen-activated protein kinase (MAPK)-dependent manner (Strickland et al., 2005). PLVAP upregulation by phorbol 12-myristate 13-acetate (PMA) required the activation of Extracellular signal-regulated kinase 1/2 (ERK1/2)-MAPK pathway (Stan et al., 2004). A disintegrin and metalloproteinase domain 10 (ADAM10)/Notch signaling could downregulate PLVAP during the development of glomeruli (Farber et al., 2018). In addition, transforming growth factor-β (TGF-β), inflammatory mediators such as tumor necrosis factor-α (TNF-α), and shear stress could regulate PLVAP expression (Wasserman et al., 2002). Our study revealed that Gsα/cAMP/CREB signaling stimulates PLVAP gene expression, which enhance our understanding of PLVAP regulation.

The endothelial Gsα deficiency or overexpression could affect CREB protein level, which was consistent with previous reports from our and other groups that Gsα deficiency in smooth muscle or cardiomyocytes resulted in CREB protein reduction (Qin et al., 2017; Yin et al., 2021). Phosphorylation at Ser133 in CREB may contribute to its protein stability (Costes et al., 2006; Mouravlev et al., 2007). The molecular mechanism about the regulation of CREB protein level and stability by Gsα needs to be further investigated.

In addition to the function of vascular tone and permeability, endothelial cells also play an important role in the regulation of tissue lipid uptake and metabolism. For example, CD36 deletion in endothelial cells could increase plasma free fatty acids (FFA) and postprandial TG levels (Son et al., 2018) and its deletion in lymphatic endothelial cells caused visceral obesity and insulin resistance (Cifarelli et al., 2021). Endothelial peroxisome proliferator activated receptor-γ (PPARγ) knockout mice exhibited increased serum FFA and TG levels, decreased adiposity and increased insulin sensitivity in responses to high-fat diet (Kanda et al., 2009). Capillary endothelial cells uptake of lipids might occur *via* caveolae-mediated transcytosis. Cav-1^−/−^ mice had high blood fatty acid (FA) and TG levels, which may be attributed to impaired transcytosis of albumin that carries FAs and other lipids (Schubert et al., 2001). Our results showed that Gsα^ECKO^ mice displayed lower serum albumin levels, which reduced the binding between lipoprotein lipase (LpL) and vascular endothelial cells, resulting in the decreased ability of TG clearance and increased blood lipid (Shearer and Kaysen, 2006). Besides, endothelial Gsα deficiency reduced white adipose deposition. These results further confirm the important role of endothelial cells in the regulation of lipid metabolism.

In conclusion, adult endothelial Gsα deficiency increased microvascular permeability, which contributed to edema, anemia, hypoproteinemia, and hyperlipoproteinemia in Gsα^ECKO^ mice. Mechanically, Gsα regulated PLVAP expression through CREB-mediated transcription. Thus, Gsα plays a vital role in regulating endothelial cell permeability, which may provide a new strategy for the treatment of endothelial permeability-related diseases.

## Data Availability

The original contributions presented in the study are included in the article/Supplementary Material, further inquiries can be directed to the corresponding author.
