# Supplementary material for: Stimulatory G-Protein α Subunit Modulates Endothelial Cell Permeability Through Regulation of Plasmalemma Vesicle-Associated Protein
Source: Front Pharmacol. 2022 Jun 3;13:941064. doi: 10.3389/fphar.2022.941064 (PMC9204201; doi:10.3389/fphar.2022.941064)
Supplement: Supplementary file 1 [file DataSheet1.DOCX]

**Supplement**

**
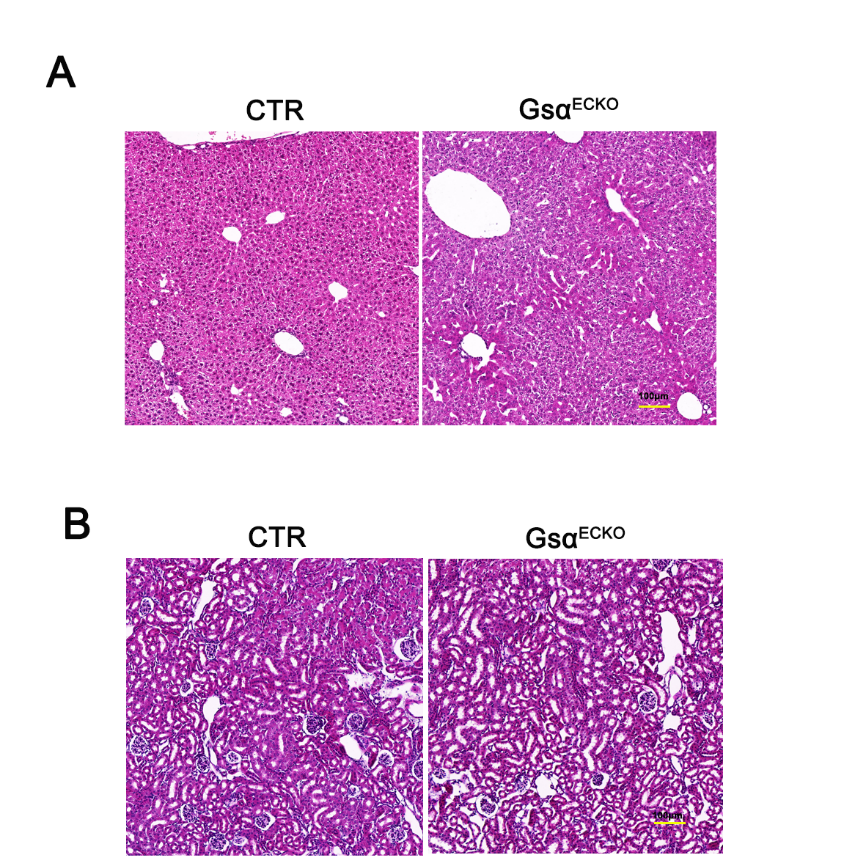
**

**Supplementary Figure 1.** Gsα^ECKO^ mice exhibited normal kidney and liver histology. **A,** H&E staining of liver tissue sections from CTR and Gsα^ECKO^ mice. Scale bar, 100 μm. **B**, H&E staining of kidney tissue sections from CTR and Gsα^ECKO^ mice. Scale bar, 100 μm.
